# The role of resveratrol, Sirtuin1 and RXRα as prognostic markers in ovarian cancer

**DOI:** 10.1007/s00404-021-06262-w

**Published:** 2021-12-06

**Authors:** Fangfang Chen, Thomas Kolben, Sarah Meister, Bastian Czogalla, Theresa M. Kolben, Anna Hester, Alexander Burges, Fabian Trillsch, Elisa Schmoeckel, Doris Mayr, Artur Mayerhofer, Sven Mahner, Udo Jeschke, Susanne Beyer

**Affiliations:** 1 Department of Obstetrics and Gynecology, University Hospital, LMU Munich, Germany, Marchioninistr. 15, 81377 Munich, Germany; 2 Institute of Pathology, University Hospital, LMU Munich, Germany, Marchioninistr. 15, 81377 Munich, Germany; 3 Biomedical Center Munich (BMC), Cell Biology, Anatomy III, LMU Munich, Großhadernerstraße 9, Planegg, Martinsried, 82152 Munich, Germany; 4 Department of Obstetrics and Gynecology, University Hospital, Universitätsklinikum Augsburg, Germany, Stenglinstr. 2, 86156 Augsburg, Germany

**Keywords:** Ovarian cancer, Survival, Epigenetics, Resveratrol, Sirtuin1, RXR

## Abstract

**Objective:**

Ovarian cancer is the most lethal gynecologic cancer. Resveratrol (RSV) is known to alter metabolism in cancer. It affects the nuclear retinoid-X-receptor (RXR), which implies a modulating effect of RXR to gynaecologic cancers. Furthermore, RSV targets Sirtuin1 (Sirt1), a histone deacetylase.

**Study design:**

123 tissue samples of patients with serous or mucinous ovarian cancer were examined for expression of Sirt1 and RXR. Ovarian cell lines were treated with RSV and consequences on viability and apoptosis were evaluated. The influence of RSV to Sirt1 and RXR expression was analyzed by western blotting

**Results:**

A correlation of nuclear Sirt1 and RXRα expression could be detected (*p* = 0.006). Co-expression of nuclear RXRα and cytoplasmic (*p* = 0.026) or nuclear (*p* = 0.041) Sirt1 was associated with significantly increased overall survival in advanced tumour stages. Viability was decreased in all cell lines after stimulation with resveratrol, while cell apoptosis was increased. RSV treatment led to significant lower Sirt1 expression in A2780 cells (*p* = 0.025) and significant increased RXR expression in cisA2780 cells (*p* = 0.012)

**Conclusion:**

In order to use RSV as medical target, studies could be developed to improve the understanding of drug resistance mechanisms and consequently improve treatment outcome.

**Supplementary Information:**

The online version contains supplementary material available at 10.1007/s00404-021-06262-w.

## Introduction

Ovarian cancer is the most lethal gynaecologic malignancy and the eighth leading cause of cancer-related mortality among women worldwide [1, 2]. The overall 5-year survival rates have barely improved over the past few decades remaining at 40–45% for advanced stages and around 80% of patients progressing within 18 months [3, 4]. The poor prognosis of ovarian cancer is mainly related to late-stage diagnosis and the rapid development of resistance to current chemotherapy regimens [2, 5].

Resveratrol (RSV), a naturally plant polyphenol originates from grapes and berries, has been proven to alter metabolism in cancer [3] and to regulate tumour microenvironment [6]. The regulatory effect of RSV on cancer is complex: besides inhibition of cell growth, RSV is also involved in enhancing chemo-sensitivity and blocking the cancer invasion of cancer cells in vitro [7]. In addition, it has been confirmed that RSV improves the efficacy of cisplatin in ovarian cancer [8].

RSV is also able to modulate vitamin D receptor (VDR)-signaling and it can induce dimerization of VDR with one of its partners, the nuclear retinoid X receptor (RXR) [9]. Three retinoic X receptors are known: RXRα [10], RXRβ [11], and RXRγ [12]. Although the distribution of RXR subtypes is different, their functions are similar: modulating gene expression they control numerous functions by dimerization with other nuclear hormone receptors, contributing thereby to activities of different cell fates [13]. As the VDR is known to be involved in gynaecologic cancers, the interaction of VDR with the RXR implies that also the RXR may have a modulating effect on gynaecological cancers [14].

Sirtuin1 (Sirt1) is a NADP-dependent histone deacetylase, which regulates cellular metabolism and cellular pathways [15–20]. Its role regarding cancer progression remains controversial as it can act as tumour suppressor or as tumour promotor [21]. In ovarian cancer, Sirt1 overexpression was correlated with improved overall survival [22]. By complex mechanisms (via interaction of NF-kB), Sirt1 can be influenced by RSV: it can be targeted by RSV, which can lead to suppression of tumourigenesis in colorectal carcinoma [23].

Recently, RSV was described to activate RXR and stimulate Sirt1 in mammalians [9]. The competitive binding of RXR and Sirt1 to PPARα (a peroxisome proliferator-activated receptor) could be due to structural similarity between these proteins. Sirt1 binds to PPARα more strongly than RXRα suggesting that Sirt1 interacts with PPARα directly rather than RXR [24]. Regnault et al. noticed that RSV induced Sirt1 and RXR in muscle hypoxia [25]. Unfortunately, the effect of RSV on Sirt1 and RXR expression in ovarian cancer cells has not been well documented until now.

In the present study, we immunohistochemically examined the expression of RXRα and Sirt1 in mucinous and serous ovarian cancer and analyzed the relationship between RSV, RXRα and Sirt1 in ovarian cancer in vitro. Considering the potential medical role of resveratrol in ovarian cancer, we evaluated the effects of RSV by proliferation and apoptosis experiments. In addition, we investigated the mechanism of RSV resistance to apoptosis in ovarian cancer cell lines. This study aimed to analyze RXRα and Sirt1 as potential therapeutic targets in ovarian cancer.

## Materials and methods

### Material

In this study, we used ovarian cancer tissue samples of 123 patients who underwent surgery for ovarian cancer from 1990 to 2002 at the Department of Gynecology and Obstetrics, Ludwig-Maximilians-University of Munich, Germany. Patients who underwent surgery due to serous or mucinous ovarian cancer were included while other histological subtypes were excluded due to low number. The median age was 59 years (range 20–88 years) and median overall survival was 2.67 years. The distribution of clinic-pathological variables can be seen in Table 1. As positive controls for immunohistochemical staining, we utilized palatine tonsil for Sirt1 staining and first trimester placenta for RXR staining, both received from the Department of Obstetrics and Gynecology of the Ludwig-Maximilians-University of Munich. Clinical and follow-up data for statistical analyses were provided by the Munich cancer registry and retrieved from medical records.

**Table 1:** Patients’ characteristics

	*N*	%
Subtype		
Serous	110	89.4
Mucinous	13	10.6
Age		
≥ 60	61	49.6
< 60	62	50.4
FIGO		
I/II	29	23.6
III/IV	92	74.8
NA’s	2	01.6
Grading		
1/2	76	61.8
3	40	32.5
NA’s	7	05.7
Progression (18 years)		
No progression	101	82.1%
Progression	21	17.1%
NA’s	1	0.1%
Overall-Survival (18 years)		
Right censured	38	30.1%
Died	84	68.3%
NA’s	1	0.1%

### Ethics approval

All ovarian cancer specimens had been collected for histopathological diagnostics during surgery. They were no longer used for clinical tests. Patients’ data were anonymized and authors were blinded for clinical information during experimental analyses. The study was conducted in consent to the Declaration of Helsinki and was approved by the local ethics committee of the Ludwig-Maximilians University of Munich (reference number 227-09 and 18-392).

### Immunohistochemistry

Paraffin-embedded slides of 3 µm were dewaxed in xylol and washed in 100% ethanol. For inhibition of the endogen peroxidases, tissue samples were incubated in 3% methanol/H_2_O_2_ and rehydrated in a descending alcohol series. Slides were afterwards heated in a pressure cooker using sodium citrate buffer (pH 6.0; containing 0.1 M citric acid and 0.1 M sodium citrate in distilled water). After cooling and washing in PBS (phosphate-buffered saline), all slides were incubated with blocking solution to avoid non-specific binding of the primary antibodies. Subsequently, the slides were stained with the primary antibodies anti-Sirt1 and anti-RXRα (Table 2) and incubated. After washing, the secondary complexes of the ABC detection kits were applied following the manufacturer’s protocols to detect reactivity. Immunostaining was visualized with the substrate and the chromogen-3, 3′-diaminobenzidine (DAB) for 1 min. For exact staining protocol, see Table 2.

**Table 2:** Antibodies and chemicals used for the immunohistochemistry

Anti-Sirt1^a^	Anti-RXRα^b^
PBS^c^	PBS^c^
Blocking solution^d^: 20 min	Blocking solution^d^: 20 min
primary antibody^a^: 1:1000 Incubation: 16 h, 4 °C min	Primary antibody^b^: 1:200 Incubation: 16 h, 4 °C
ABC detection kid^e^	ABC detection kid^e^
Chromogen: DAB^e^ (1 min)	Chromogen: DAB^e^ (1 min)

^a^Anti-Sirt1 rabbit IgG, polyclonal antibody, concentration: 1:1000; Atlas Antibody, Sweden; order number: SHPA006295

^b^Anti-RXRα rabbit IgG, polyclonal antibody, concentration: 1:200; PPMX, Japan; order number: pp-k8508-10

^c^HRP-Polymer-Kit (mouse/rabbit); Zytomed Systems, Germany; order number: POLHRP-100

^d^ABC detection kid; Vectastain, USA; order number: AK-6401

^e^Dulbecco’s phosphate buffered saline; Gibco, USA; order number: 14190-094

For the light microscopy analysis, the semi-quantitative immune-reactive score (IRS) is calculated via the multiplication of optical staining intensity (grades: 0 = no, 1 = weak, 2 = moderate and 3 = strong staining) and the percentage range of positive stained cells (0 = no staining, 1 =  ≤ 10% of the cells; 2 = 11–50% of the cells; 3 = 51–80% of the cells and 4 =  ≥ 81% of the cells were stained for the antibody, respectively). Palatine tonsil was used as control for Sirt1 and first trimester placenta was used as control for RXR staining.

### Cell culture

Human ovarian cancer cell lines with different characteristics (A2780, UWB1.289 and cisA2780, see Table 3) were used in the study. The cell lines were ordered from Gibco (see Table 3).

**Table 3:** Cell lines

	A2780^a^	UWB1.289^b^	cisA2780^c^
Cell type	Epithelial ovarian cancer cell	Epithelial ovarian cancer cell	Epithelial ovarian cancer cell
Characteristics	Mucinous	Serous brca1-null	Mucinous, carboplatin-resistant
Culture medium	RPMI 1640^d^ + 10% FBS^e^	RPMI 1640^d^ + 10% FBS^e^	RPMI 1640^d^ + 10% FBS^e^

^a,b,c^Gibco

^d^Gibco, USA; Order number: 21875-034

^e^Foetal Bovine Serum, biochrom, Germany; order number S0615

### Assays

#### Cell viability assay

A2780, UWB1.289 and cisA2780 ovarian cancer cells were seeded at the density of 1.5 × 10^4^ cells/well in 96 well plates with 200 μl medium. After 20 h cell culture medium was replaced with fresh culture medium with 50 µM and 100 µM of resveratrol (RSV; Sigma, America; order number: R5010-100MG) for 24 h. Untreated control cells were plated in medium only. To each well, 20 μg MTT (Sigma, USA; order number: M-5655) were added for 1.5 h at 37 °C to show viability. After removing MTT from the plates, 200μL DMSO (dimethyl sulfoxide; concentration: 0.5%; SERVA, Germany; order number: 20385, 0.5%) were added and mixed thoroughly on the shaker for 5 min at room temperature. The optical density was examined at 595 nm using Elx800 universal Microplate Reader. Each experiment was carried out in triplicate.

#### Marker of proliferation: BrdU

To confirm the results, we used BrdU-Assay, which is more sensitive. A2780, UWB1.289 and cisA2780 ovarian cancer cells were cultured at the density of 1.0 × 10^4^ cells/well together with various dilutions (50/100 µM) of resveratrol in 96-well plates. For the labelling of DNA replication BrdU (Bromodeoxyuridine; Roche, Switzerland; order number: 11647229001) was added to the culture medium for 2 h. The final concentration of BrdU was 10 μM. After the removal of BrdU by pipette, 200 µl/well FixDenat (Bromodeoxyuridine; Roche, Switzerland; order number: 11647229001) were added and cells were incubated for 30 min at room temperature. Afterwards, FixDenat solution had to be removed thoroughly and 100 µl/well anti-BrdU-POD (Bromodeoxyuridine; Roche, Switzerland; order number: 11647229001) working solution were added. Cells were then incubated for approximately 90 min at room temperature and washed 3 times with PBS. 100 µ/well substrate solution BrdU was added and incubation for 20 min was performed. To each well 25 µl 1 M H_2_SO_4_ were added and the absorbance of the samples was measured by an ELISA reader at 450 nm.

##### TUNEL

Terminal deoxynucleotidyl transferase (TdT) dUTP Nick-End Labeling (TUNEL) assay has been designed to detect apoptotic cells that undergo extensive DNA degradation during the late stages of apoptosis. TUNEL staining was performed to assess in situ DNA fragmentation using a commercial kit (FragELTM DNA Fragmentation Detection Kit, Colorimeric-TdT Enzyme, USA; order number: Qia33-1EA) following the manufacturer’s protocol.

#### Apoptosis assay

As a more specific method, Caspase assay was used to confirm the results. Hereby apoptosis was evaluated by measuring the level of caspase-cleaved cytokeratin 18 (M30, Roche, Switzerland; order number: 121140322001). The ovarian cell lines A2780, cisA2780 and UWB1.289 were seeded at a density of 1.0 × 10^4^ cells/well on 96-well plates in 200 μl medium. After 20 h, 50 or 100 µM RSV were added and cells were incubated for 24 h. M30 CytoDeath (Roche, Switzerland; order number: 121140322001, dilution 1:1000) was used to detect the apoptotic cells.

#### Western blotting

Cell lysates were extracted from A2780, cisA2780 and UWB1.289 cells with radio-immuno-precipitation assay buffer (RIPA, Sigma-Aldrich, St. Luis, USA; order number: R0278-50ML). For Western blotting, 20 µg of cell lysates was first separated in 10% sodium dodecyl sulphate–polyacrylamide gel electrophoresis and then transferred to a polyvinylidene fluoride membrane. The membrane was blocked in 10% casein and then incubated with the primary antibodies for 16 h at room temperature. We used the same antibodies as for immunohistochemistry (see Table 2).

GAPDH was used as a housekeeping gene and mouse monoclonal anti-GAPDH antibody (GeneTex, America; order number: GTX277408) was diluted 1:1000 in 10% CASEIN (Vector, Germany; order number: ZE0925). Afterwards, the membrane was incubated with the goat–anti-rabbit secondary antibody (Vector; bioZol; Germany; order number: VEC-BA-1000, dilution 1:1000) conjugated with alkaline phosphatase and detected with 5-bromo-4-chloro-3′-indolylphosphate/nitro-blue tetrazolium (BCIP/NBT)-chromogen substrate solution (Vector; bioZol; Germany; order number: Vec-SP-5020). Western blots were scanned and quantified using the GelScan V6.0 1D Analysis Software (SERVA, Electrophoresis GmbH, Heidelberg, Germany). Band intensities of Sirt1 and RXRα were normalized with band intensities of GAPDH. The blots were repeated three times.

### Statistics

SPSS Statistics 25 was used for data collection, processing and analysis. The Wilcoxon’s test was used for the evaluation of Sirt1, RXR and GAPDH values between related groups. Spearman’s test was applied to compare the IRS of Sirt1 and RXR staining in the ovarian cancer patients. Survival rates were shown by Kaplan–Meier curves. *p* value < 0.05 was considered as statistically significant.

## Results

### Correlation of RXRα and Sirt1 expression with Clinical and Pathological Data

Sirt1 and RXRα expression was analyzed in 123 cases of ovarian cancer (110 serous and 13 mucinous cases) (Table 1).

**Fig. 1:**
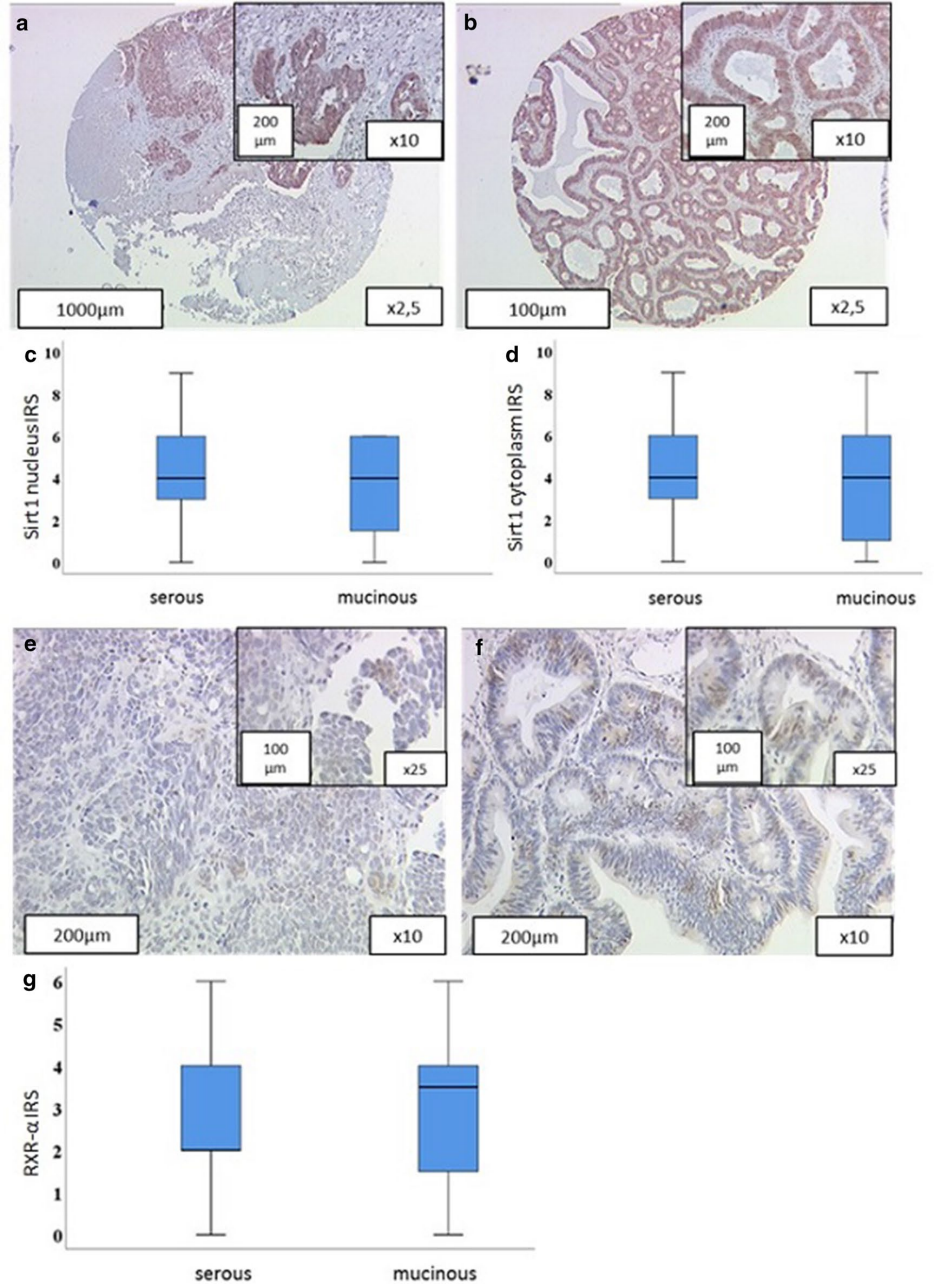
Representative immunohistochemistry images of Sirt1 and RXRα in the same view of ovarian cancer samples. **a** Sirt1 expression in serous ovarian cancer on a TMA (tissue micro array) with a 2.5 magnification and an insert at 10 × magnification. **b** Sirt1 expression in mucinous ovarian cancer on a TMA with a 2.5 magnification and an insert at 10 × magnification. **c**, **d** Boxplot: Sirt1 expression in the nucleus (**c;** *p* = 0.639**)** and in the cytoplasm (**d**; *p* = 0.915**)** with a median IRS of 4 in mucinous and serous ovarian carcinoma. **e** RXRα expression in serous ovarian cancer with a 10 × magnification and an insert at 25 × magnification. **f** RXRα expression in mucinous ovarian cancer with a 10 × magnification and an insert at 25 × magnification. **g** boxplot: RXRα expression with a median IRS of 2 and 3.5 in mucinous and serous ovarian carcinoma on slides (*p* = 0.424)

Sirt1 expression was distinguished into cytoplasmic and nuclear staining (Table 4). Cytoplasmic and nuclear Sirt1 expression was detectable in 115 cases (93.5%). 8 cases (6.5) did not express Sirt1 in the cytoplasm and the nucleus. Median IRS was 4. In the examined subcategories (mucinous, serous, high grade, low grade and different FIGO stages; Table 4; Fig. 1A–D) the median IRS was also 4 for both, nuclear and cytoplasmic expression. No significant differences regarding histological subtype (*p* = 0.915), FIGO stage (*p* = 0.568) or grading (*p* = 0.076) in cytoplasmic as well as in nuclear expression (histology: *p* = 0.639; FIGO: *p* = 0.408; Grading: *p* = 0.514) were found (Table 4). High cytoplasmic Sirt1 expression (IRS ≥ 4) was detectable in 75 cases (61.0%), 48 cases had an IRS < 4 (low IRS; 39.0%). Increased nuclear expression with an IRS ≥ 4 was found in 82 cases (high; 66.7%). 41 cases had a nuclear Sirt1 IRS smaller than 4 (low; 33.3%).

**Table 4:** Expression profile of RXRα and Sirt1 staining regarding clinical and pathological characteristics

	Sirt1 cytoplasm		Sirt1 nucleus		RXRα Nucleus	
	Median (± SD)	*p*	Median (± SD)	*p*	Median (± SD)	*p*
Histology		0.915		0.639		0.424
Serous	4 (± 1.94)		4 (± 1.81)		2 (± 0.15)	
Mucinous	4 (± 3.23)		4 (± 2.39)		3.5 (± 0.50)	
FIGO		0.568		0.408		0.405
I/II	4 (± 2.43)		4 (± 1.78)		2 (± 1.48)	
III/IV	4 (± 1.98)		4 (± 1.91)		2 (± 1.55)	
Grading		0.076		0.514		0.309
G1/G2	4 (± 2.20)		4 (± 1.96)		2 (± 1.394)	
G3	4 (± 1.50)		4 (± 1.82)		2 (± 1.773)

A total of 114 cases of ovarian cancer expressed RXRα (see Table 4) in the nucleus with a median of 2, 4 did not express RXRα at all and 5 cases were not evaluable. Cytoplasmic RXRα staining was not detectable. High RXRα expression (IRS ≥ 3) was detectable in 44 cases (35.7%) as compared to low expression (IRS < 3) in 74 cases (60.2%). Analysis of the correlation between RXRα expression and histopathological parameters revealed: median IRS in serous specimens was 2 (SD ± 0.147) compared to a median IRS of 3.5 in mucinous carcinomas (SD ± 0.499; *p* = 0.424; Table 4; Fig. 1E–G). Regarding the grading and FIGO, the median IRS was 2 in high grade and low grade cancers (*p* = 0.309) as well as in different FIGO stages (*p* = 0.405).

A significant positive correlation between nuclear Sirt1 and RXRα in IRS staining was detected using Spearman’s test (*p* = 0.006; Table 5).

**Table 5:** Spearman’s correlation analysis between SIRT 1 and RXRα

	Sirt1nucleus	Sirt1cytoplasm
RXRα		
Correlation coefficient	− 0.259	0.163
*p*	**0.006**	0.085

Significant results (*p* < 0.05) are shown in bold

**Table 6:** Multivariate analysis

Covariate	Coefficient (Bi)	Exp(*B*)	95%CI		*p* value
			Lower	Upper	
Subtype	0.109	1.115	0.642	1.937	0.699
FIGO	1.327	3.771	1.956	7.271	**0.000**
Grade (I/II vs. III/IV)	− 0.604	0.547	0.355	0.843	**0.006**
Age (< 60 vs. ≥ 60 years)	0.359	1.432	0.944	2.170	0.091
Sirt1cytoplasm	0.004	1.004	0.868	1.161	0.959
Sirt1nucleus	0.035	0.965	0.821	1.135	0.670
RXRα nucleus	− 0.096	0.908	0.908	1.057	0.213

Prognostic impact of Sirt1 cytoplasm (*p* = 0.959), Sirt1-nucleus (*p* = 0.670) and RXRα nucleus (*p* = 0.213) alone was not significant (Table 6)

Significant results (*p* < 0.05) are shown in bold

**Fig. 2:**
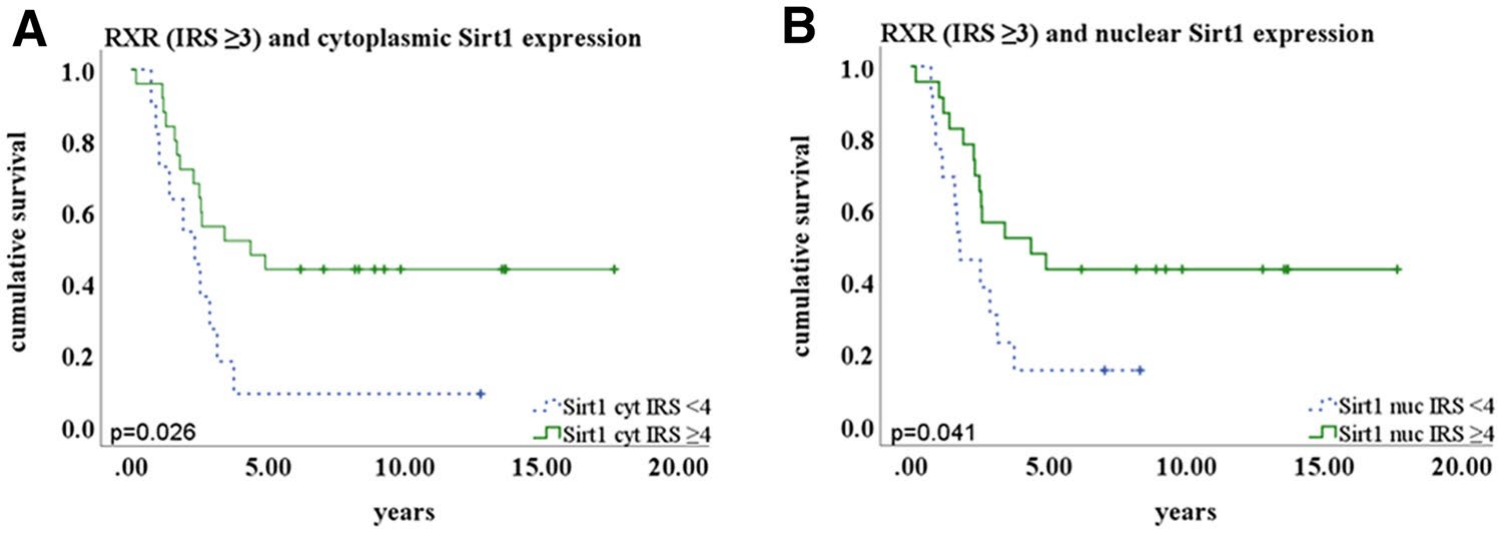
Overall survival in patients with cytoplasmic Sirt1expression (**A;** *p* = 0.026) and overall survival in patients with nuclear RXRα expression (**B;** *p* = 0.041). Statistical significance for all tests was assumed for *p* < 0.05

As shown in the Kaplan–Meier curve (Fig. 2), co-expression of Sirt1 and nuclear RXRα was associated with significant longer survival time after diagnosis in advanced tumour stages (FIGO III/IV). This is significant for cytoplasmic Sirt1 expression (*p* = 0.026; Fig. 2a) as well as for nuclear Sirt1 expression (*p* = 0.041; Fig. 2b).

### Correlation of resveratrol to apoptosis of ovarian cancer

**Fig. 3:**
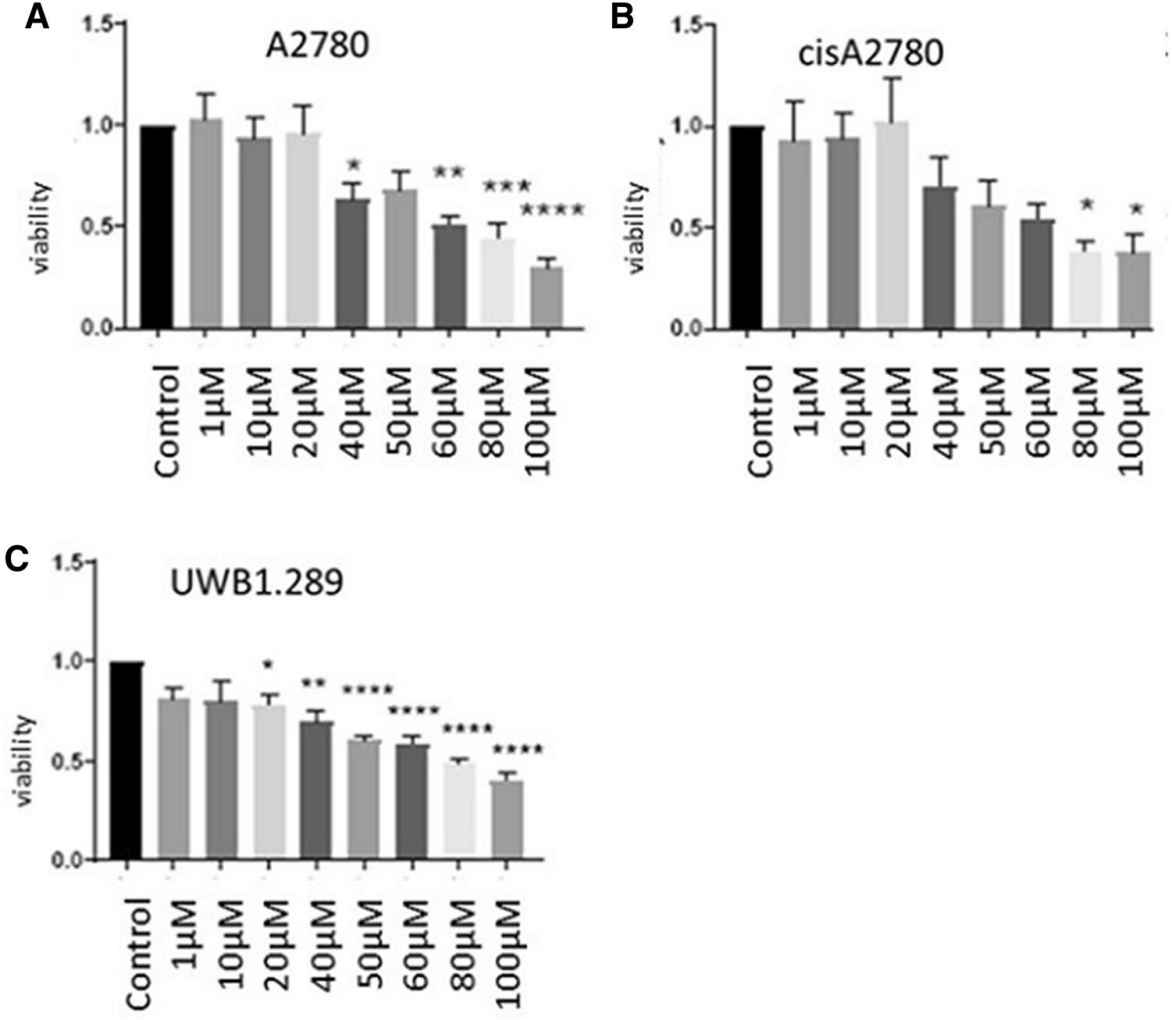
Cytotoxity of RSV: ovarian cancer cell lines were treated with RSV (50 µM and 100 µM) for 24 h. The cell viability was determined with MTT assay. **a** A2780 (*A2780 control vs. RSV 40 µM p = 0.0032; **A2780 control vs RSV 60 µM *p* = 0.002; *** A2780 control vs. RSV 80 µM *p* = 0.0004; ****A2780 control vs. RSV 100 µM *p* < 0.0001), **b** cisA2780 (*cisA2780 control vs RSV 80 µM/RSV 100 µM *p* < 0.0001) and **c** UWB1.289 (*UWB1.289 control vs. RSV 20 µM *p* = 0.0326; **UWB1.289 control vs. RSV 40 µM *p* = 0.0013; ****UWB1.289 control vs. RSV 50/60/80/100 µM *p* < 0.0001). The data are presented as the means ± SEM. *N* = 3. *p < 0.05

**Fig. 4:**
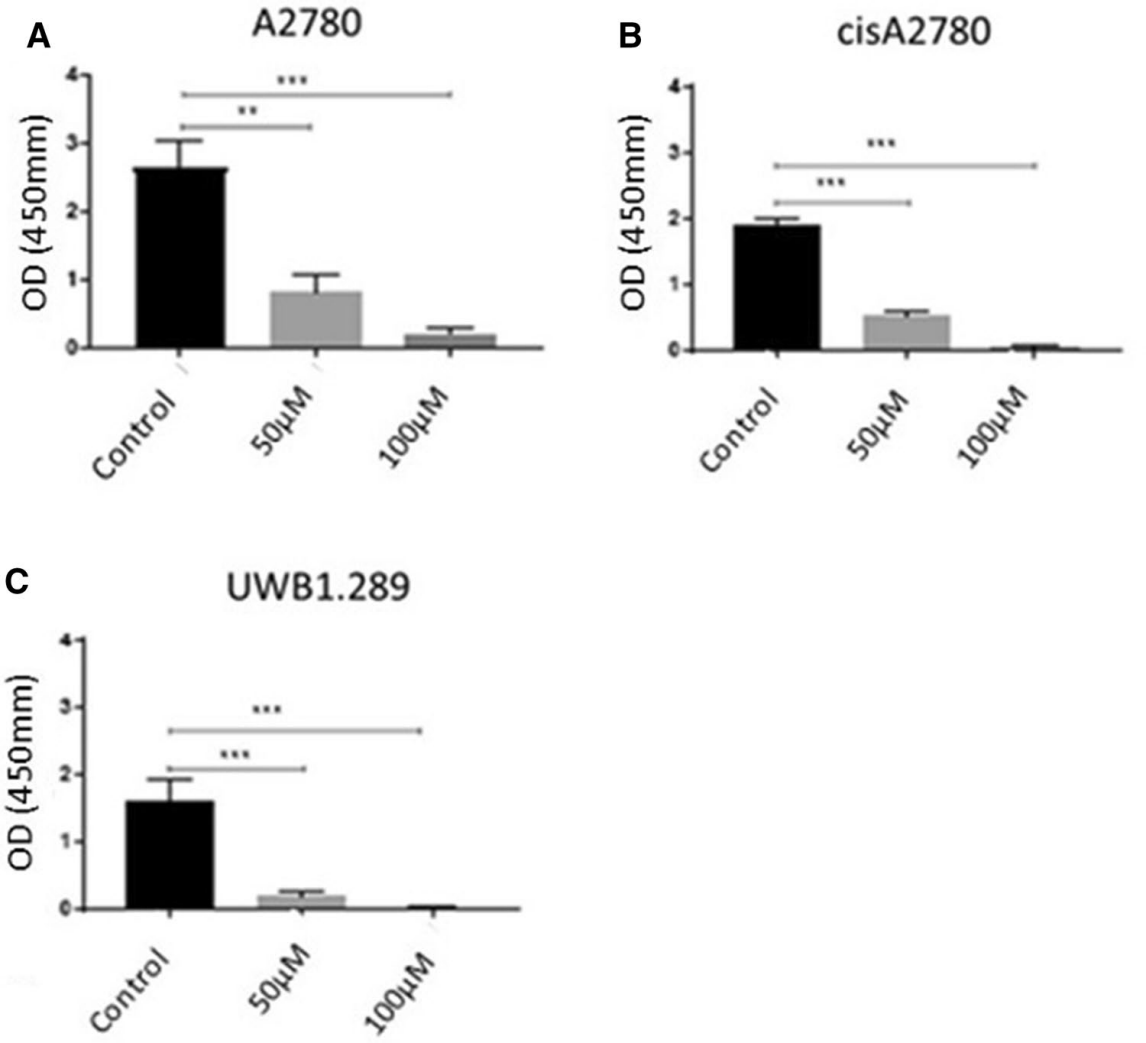
Cells were treated with RSV (50 µM and 100 µM) for 24 h and BrdU (final concentration is 10 µM) was added. BrdU in corporation was determined by measuring the absorbance at 450 nm. **A** A2780 (**A2780 control vs. RSV 50 µM *p* = 0.0019; ***A2780 control vs. RSV 100 µM *p* = 0.003); **B** cisA2780 (**cisA2780 control vs. RSV 50/100 µM *p* < 0.0001); **C** UWB1.289 (***UWB1.289 control vs. RSV 50 µM *p* = 0.0007; ***UWB1.289 control vs. RSV 100 µM *p* = 0.0003). Representative results are presented as the means ± SEM. (*N* = 3) *p < 0.05

**Fig. 5:**
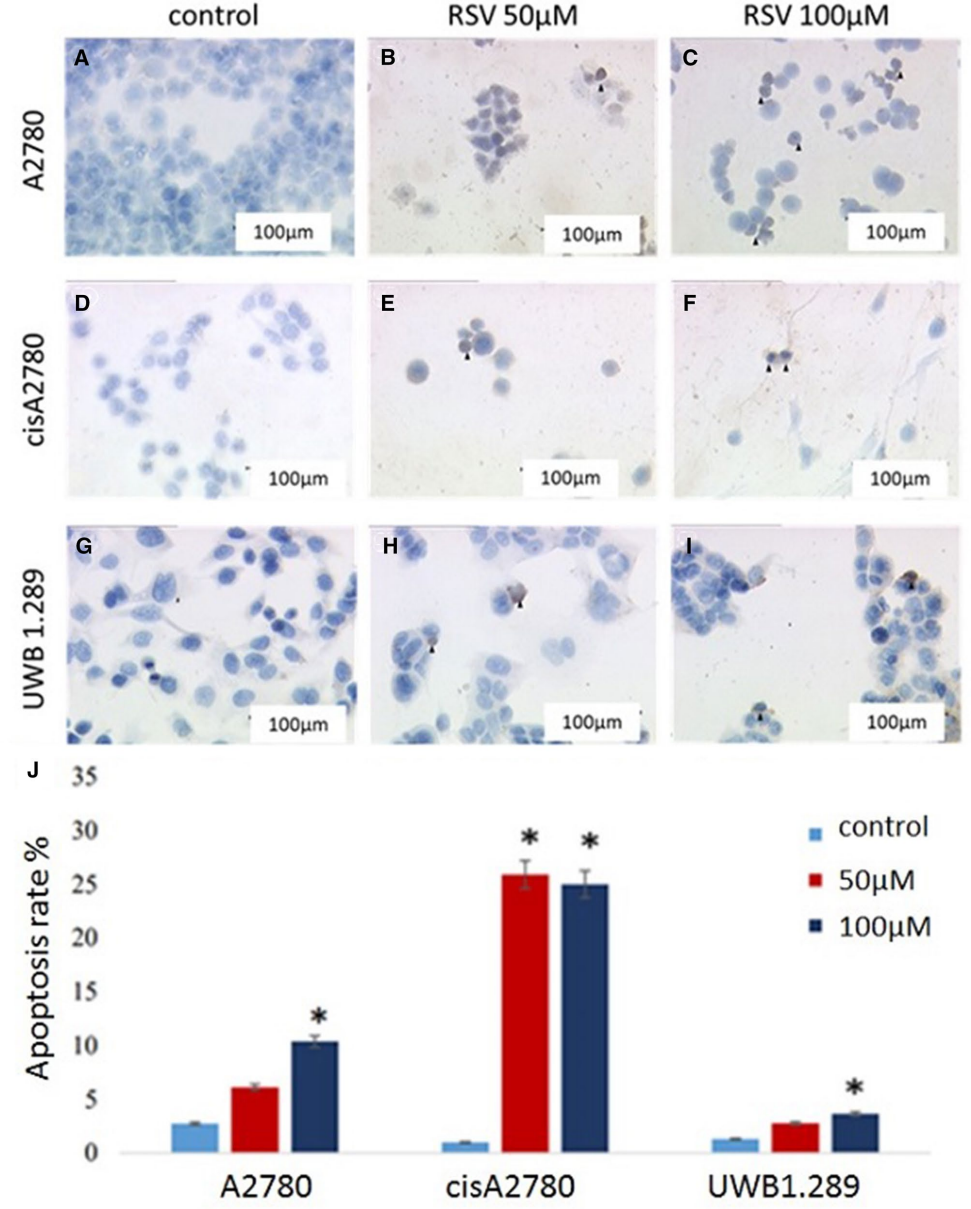
The effects of RSV-treatment and M30 identification on A2780 (**A**–**C**), cisA2780 (**D**–**F**) and UWB1.289 (**G**–**I**) cells with 50 µM and 100 µM resveratrol for 24 h. (*N* = 5). Apoptosis rates in dependent of RSV concentration are shown in a boxplot (**J**). The data are presented as means ± SEM. *p < 0.05

The results of the MTT assay showed that viability was decreased in all cell-lines (A2780, cis A2780 and UWB1.289) after stimulation with resveratrol (RSV). This effect was dose-dependent (Fig. 3). Cell apoptosis, measured via BrdU assay, indicated that the apoptotic features were obviously improved in 100 μM RSV treated cells (Fig. 4, *p* < 0.003), meaning that apoptosis was increased. Furthermore, cell morphology observation showed a change in apoptotic markers (the brown cytoplasm, marked by M30): apoptosis rate was significantly increased in A2780 cells and UWB1.289 treated with resveratrol 100 µM (*p* = 0.043; Fig. 5) compared to the control. In A2780cis, M30 was significantly increased in cells treated with RSV 50 µM and RSV 100 µM in comparison with the control (*p* = 0.042 and 0.043; Fig. 5).

**Fig. 6:**
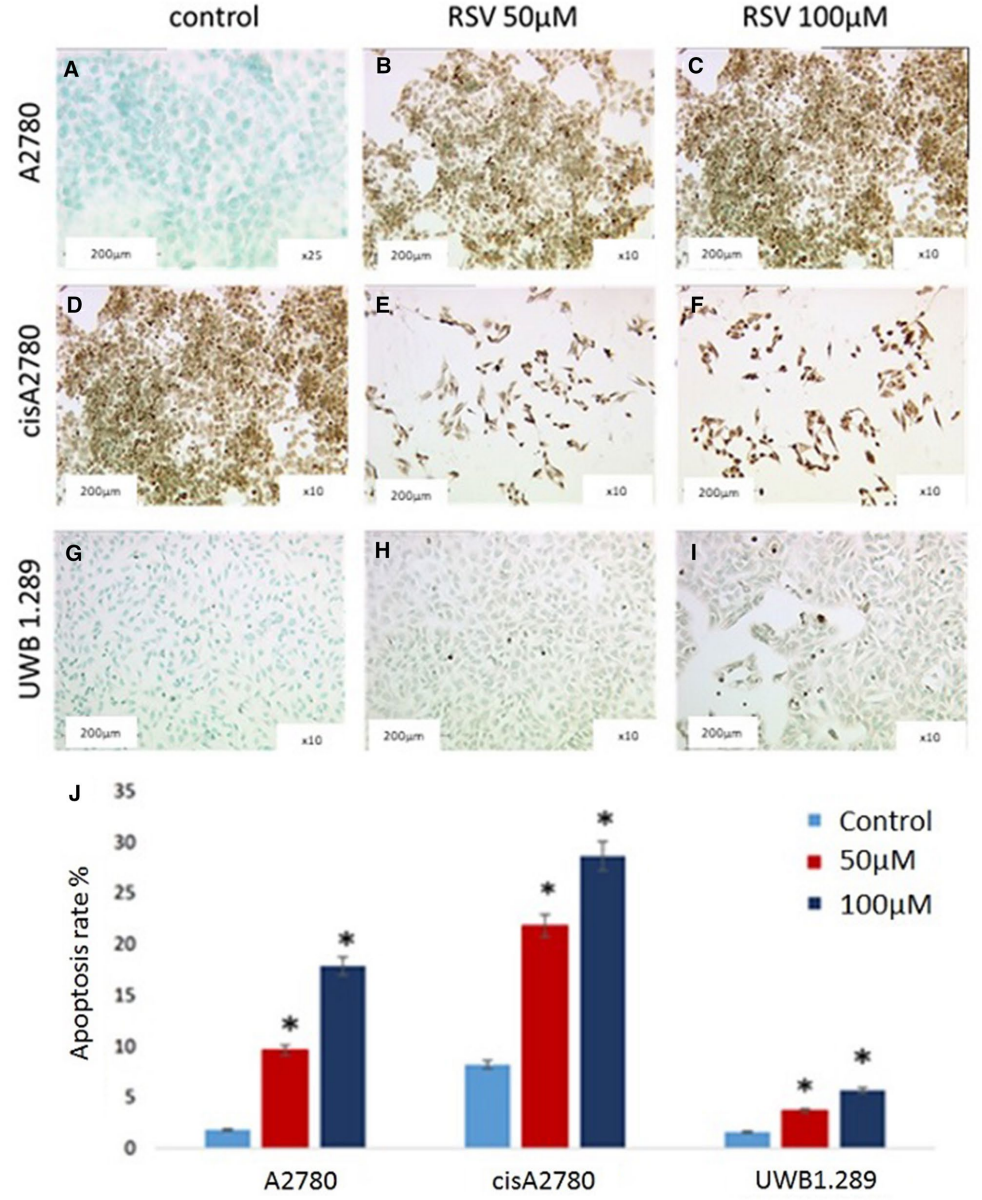
The apoptosis of A2780 (**A**–**C**), cisA2780 (**D**–**F**) and UWB1.289 (**G**–**I**) were determined by TUNEL assay. All images are at 2.5 × magnification with an insert at 10 × magnification. Apoptosis rates in dependence of RSV concentration are shown in a boxplot (**J**). The data are presented as means ± SEM. (*N* = 5) *p = 0.043

After RSV treatment, the percentage of TUNEL stained cells increased, meaning that apoptosis rate increased (Fig. 6; *p* = 0.043).

### The relationship between Sirt1 and RXRα

**Fig. 7:**
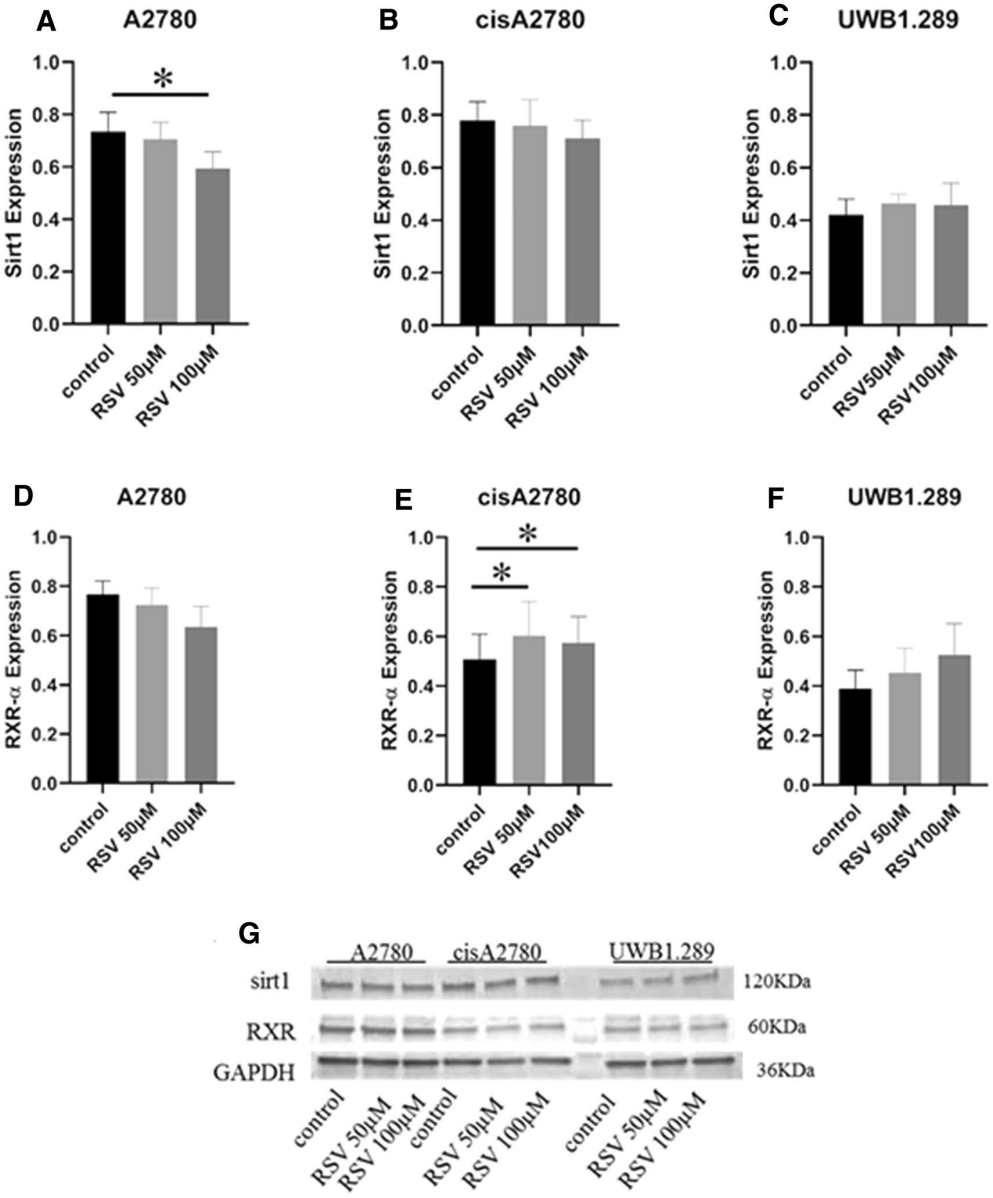
Ovarian cancer cells were treated with resveratrol 50 µM and resveratrol 100 µM for 24 h. Expression of Sirt1 in A2780 cells (**A**), cisA2780 (**B**) and UWB1.289 (**C**) cell-lines. RXRα expression in A2780 (**D**), cisA2780 (**E**) and UWB1.289 (**F**) cell-lines after RSV treatment. Finally, expressions were analyzed by western blotting (**G**). Representative results are presented as the means ± SEM. *p < 0.05

We tested the involvement of Sirt1 and RXRα in RSV-induced apoptosis in ovarian cancer cell lines. Sirt1 expression was significant lower in A2780 cells treated with resveratrol 100 µM (*p* = 0.025; 50 µM: *p* = 0.208; Fig. 7A) as compared to its control. No significant difference in the Sirt1 expression after RSV-treatment was found in cisA2780 (*p* = 0.327 and 0.069; Fig. 7B) and UWB1.289 (*p* = 0.401 and 0.575; Fig. 7C). RXRα expression was significantly increased in cisA2780 cells treated with RSV 50 µM and RSV 100 µM in comparison with the control on protein level (*p* = 0.012 and 0.017; Fig. 7E). In A2780 (*p* = 0.208 and 0.069) and UWB1.289 cells (*p* = 0.093 and 0.069) treated with RSV 50 µM or RSV 100 µM for 24 h, no significant differences were found as compared to their controls (Fig. 7D, F). Expressions were analyzed by western blot (Fig. 7G).

## Discussion

Our report shows that the expression of nuclear RXRα and Sirt1 in advanced ovarian cancer is significantly associated with longer overall survival. Resveratrol could reduce the proliferation and even increase apoptosis of ovarian cancer cells. On protein level, resveratrol (100 µM, 24 h) upregulated the expression of RXRα in the carboplatin-resistant cell-line cisA2780 and downregulated Sirt1 expression in A2780.

Current studies focus on RSV, a naturally plant polyphenol which is able to inhibit cell growth. Furthermore, it was shown to enhance chemo-sensitivity and to stop cancer invasion [7]. In addition, RSV was observed to induce apoptosis in ovarian cancer cells [26, 27]. Pizarro et al. reported that 100 µM RSV reduced cell viability and caused apoptosis after 24 h of treatment in neuroblastoma cells. In our experiment, we used the same concentration and treatment. We could confirm that RSV partially blocked cell proliferation and induced apoptosis in all examined ovarian cancer cell lines. The effect of apoptosis seems to be synergistic to cisplatin [28]. In addition, the recent studies have demonstrated that RSV (200 μM/48 h) promoted an excessive cellular ROS (2–3 times) production which induced cellular death [29]. With reference to this study, it may also be of significance that the used concentrations were higher and time of RSV exposition was longer (200–500 µM, 48 h) as compared to our study design.

Sirt1, a member of the Sirtuin family, is a NADP-dependent histone deacetylase and has a conserved catalytic core domain. Sirt1 regulates cellular defense and cell fate [15–20]. It has been considered to act dualistically either suppressing or promoting cancer, depending on the temporal and special distribution of different Sirt1 upstream and downstream factors [30]. As Sirt1 is described to induce chemo-resistance and to be associated with poor prognosis in ovarian cancer [31–34], we intended to analyze treatment with RSV in regard to Sirt1. Exposure to RSV was correlated with decreased Sirt1 expression in mucinous ovarian cancer cell-lines (A2780). Pizarro et al. determined that the decrease of Sirt1 stimulated by RSV is not responsible for apoptosis induction [35]. Based on these results, Sirt1 inhibitors could not change cell viability or apoptosis rates [35]. Bjorklund et al. showed that the RSV induced potentiation of platinum drugs in ovarian cancer was not correlated to the Sirt1 1 level by using RSV-concentrations up to 40 µM [36]. Our results contrast these findings. However, in our experiments higher concentrations of RSV were used. Nevertheless, these findings seem not to be transferable to all ovarian cancer cells, as RSV did not decrease Sirt1 expression in carboplatin-resistant cell lines. This finding has to be explored more accurately in further experiments since this patient group still suffers from very poor survival rates.

Sirt1 affects many nuclear receptors. Some of them, for example VDR, need the RXR for dimerization [37–39]. RXR plays a critical role in mediating ovarian cancer growth suppression [40]. Recent studies have demonstrated that RSV can either bind to RXR directly or modulate RXR dimerization [9]. Wang et al. also reported that RARα/RXR synergism prompt apoptosis and dampened cell proliferation [41]. Further reports showed that overexpression of RXRα could promote tumour growth by interacting with tumour necrosis factor-alpha-induced phosphoinositide 3-kinase and NF-κB signal transduction pathways [42]. In addition, a recent study showed that the “rexinoid apoptosis” involves activation of both iNOS and eNOS by RXR-PPARgamma, resulting in the production of apoptogenic NO, which induced cell apoptosis [43]. In the present study, we evaluated RXRα inhibited resveratrol-stimulated apoptosis of ovarian cancer cells. Our results suggest that RXRα could play an important role in the regulation of apoptosis in human ovarian cancer.

Increased expression of RXRα and Sirt1 was associated with increased survival rates in advanced stages of ovarian cancer. Little data exist about RXRα in ovarian cancer. But it is well known, that its stimulation with retinoids and a high amount of RXRα lead to an inhibition of tumour growth [40]. In contrast, an overexpression of Sirt1 in ovarian cancer is associated with poor prognosis [31]. In our panel, the expression of RXRα seem to neutralize this effect. It has to be examined, if this effect can be even improved by stimulation of RXRα with retinoids.

RSV seems to be an excellent candidate for potentiation of platinum treatment and to induce apoptosis in ovarian cancer. Nevertheless, these findings have to be confirmed in a larger number of specimens. Therefore, further investigations focusing on RSV and its role in anticancer effect in combination with platinum is warranted.

## Conclusion

In conclusion, we observed that the combination of nucleus RXRα and Sirt1 expression was correlated with increased overall survival in late-stage ovarian cancer. RSV, which induces apoptosis and decreases proliferation in human ovarian cancer cell lines, was associated with decreased expression of Sirt1 in mucinous ovarian cancer and increased expression of RXRα in mucinous, carboplatin resistant ovarian cancer cells. Novel strategies should be developed in order to improve the understanding of drug resistance mechanisms and to improve medical treatment. Undoubtedly, new studies of ovarian cancer for efficient, rapid and effective treatments are required.

## Supplementary Information

Below is the link to the electronic supplementary material.

Supplementary Figure X Westernblot with the represent result of Cell-linesA2780, cisA2780 and UWB1.289 after treatment with RSV for 24 hours. The samples derive from the same experiment. The images multiple exposure. Western blots were scanned and quantified using the GelScan V6.0 1D Analysis Software (SERVA, Electrophoresis GmbH, Heidelberg, Germany)

## Abbreviations

BrdU: Bromodeoxyuridine
IRS: Immunoreactivity score
PPARα: Peroxisome proliferator-activated receptor alpha
RSV: Resveratrol
RXR: Retinoid X receptor
Sirt1: Sirtuin 1
TUNEL: Terminal deoxynucleotidyl transferase dUTP Nick-End Labelling
VDR: Vitamin D receptor
