# Supplementary figures and images for: The role of resveratrol, Sirtuin1 and RXRα as prognostic markers in ovarian cancer
Source: Arch Gynecol Obstet. 2021 Dec 6;305(6):1559–72. doi: 10.1007/s00404-021-06262-w (PMC9166836; doi:10.1007/s00404-021-06262-w)

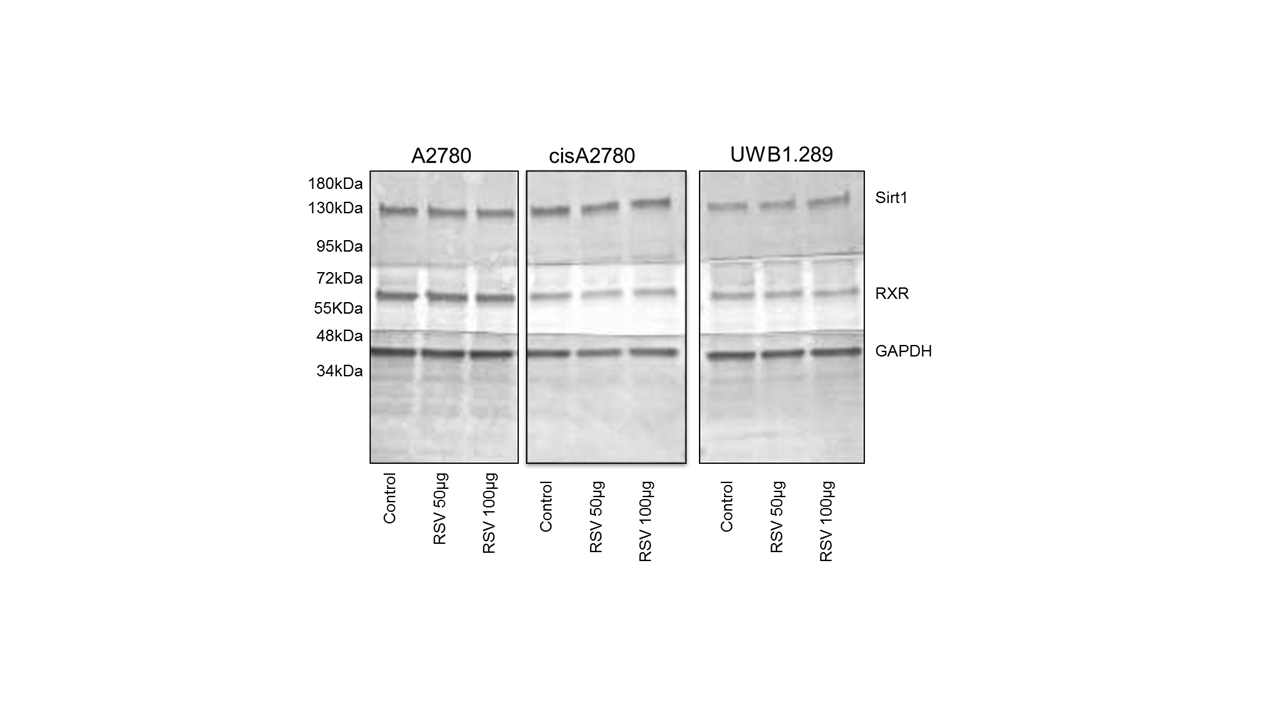

Supplement: Supplementary file 1 — Supplementary Figure X Westernblot with the represent result of Cell-linesA2780, cisA2780 and UWB1.289 after treatment with RSV for 24 hours. The samples derive from the same experiment. The images multiple exposure. Western blots were scanned and quantified using the GelScan V6.0 1D Analysis Software (SERVA, Electrophoresis GmbH, Heidelberg, Germany) [file 404_2021_6262_MOESM1_ESM.tif]
